# Epigenomic map of human liver reveals principles of zonated morphogenic and metabolic control

**DOI:** 10.1038/s41467-018-06611-5

**Published:** 2018-10-08

**Authors:** Mario Brosch, Kathrin Kattler, Alexander Herrmann, Witigo von Schönfels, Karl Nordström, Daniel Seehofer, Georg Damm, Thomas Becker, Sebastian Zeissig, Sophie Nehring, Fabian Reichel, Vincent Moser, Raghavan Veera Thangapandi, Felix Stickel, Gustavo Baretton, Christoph Röcken, Michael Muders, Madlen Matz-Soja, Michael Krawczak, Gilles Gasparoni, Hella Hartmann, Andreas Dahl, Clemens Schafmayer, Jörn Walter, Jochen Hampe

**Affiliations:** 1 Medical Department 1, University Hospital Dresden, Technische Universität Dresden (TU Dresden), Dresden, Germany; 2 Center for Regenerative Therapies Dresden (CRTD), Technische Universität Dresden (TU Dresden), Dresden, Germany; 3 Department of Genetics and Epigenetics, Universität des Saarlandes, Saarbrücken, Germany; 4 Department of Visceral Surgery, University Hospital Schleswig-Holstein, Christian-Albrechts-University Kiel, Kiel, Germany; 5 Department of Hepatobiliary Surgery and Visceral Transplantation, University of Leipzig, Leipzig, Germany; 6 Department of Gastroenterology, University of Zürich, Zürich, Switzerland; 7 Institute of Pathology, University Hospital Dresden, Technische Universität Dresden (TU Dresden), Dresden, Germany; 8 Institute of Pathology, University Hospital Schleswig-Holstein, Christian-Albrechts-University Kiel, Kiel, Germany; 9 Rudolf-Schönheimer-Institute for Biochemistry, University of Leipzig, Leipzig, Germany; 10 Institute of Medical Informatics and Statistics, Christian-Albrechts University, Kiel, Germany; 11 Center for Molecular and Cellular Bioengineering (CMCB), Technische Universität Dresden (TU Dresden), Dresden, Germany

## Abstract

A deeper epigenomic understanding of spatial organization of cells in human tissues is an important challenge. Here we report the first combined positional analysis of transcriptomes and methylomes across three micro-dissected zones (pericentral, intermediate and periportal) of human liver. We identify pronounced anti-correlated transcriptional and methylation gradients including a core of 271 genes controlling zonated metabolic and morphogen networks and observe a prominent porto-central gradient of DNA methylation at binding sites of 46 transcription factors. The gradient includes an epigenetic and transcriptional Wnt signature supporting the concept of a pericentral hepatocyte regeneration pathway under steady-state conditions. While donors with non-alcoholic fatty liver disease show consistent gene expression differences corresponding to the severity of the disease across all zones, the relative zonated gene expression and DNA methylation patterns remain unchanged. Overall our data provide a wealth of new positional insights into zonal networks controlled by epigenetic and transcriptional gradients in human liver.

## Abstract (web summary)

Spatial mapping of genomic programs in tissue cells is an important step in the understanding of organ function and disease. Here, the authors provide a spatially resolved epigenomic and transcriptomic map of human liver and show porto-central gradients in metabolic and morphogen networks and transcription factor binding sites as a basis to better understand liver regeneration and function.

## Introduction

Tissues are composed of heterogeneous cell populations, each contributing to the overall performance of the biological system. Within these populations, even morphologically similar cells of the same type may perform different functions. Deciphering this cellular heterogeneity on a functional level is key to understand tissue function and its alterations in disease. Here, functional genomics may play an important role if applied in a tissue context^1^, as combined transcriptional and epigenetic analysis provides deep insights into functional state and genomic programming of cells^2–4^. Such regionalized genomic maps may be generated by single cell analysis of isolated cells and subsequent mapping on tissue architecture using marker genes^5,6^ or by analysis of cells obtained directly from tissue context using laser capture cryo microdissection (LCM). Advances in the respective protocols allow the generation of transcriptomes^7^ and reduced representation bisulfide sequencing (RRBS) methylomes^8^ from the 100 to 200 cells obtained typically by LCM. Such analysis could provide a combined epigenetic dataset, preserve tissue context, avoid isolation procedures and may thus offer new opportunities for the analysis of shock frozen human tissue.

Despite their rather uniform morphological appearance, hepatocytes in mammalian liver are characterized by a heterogenous enzyme distribution along the porto-central axis^9,10^ which is termed metabolic zonation^11,12^. Recently, using single cell mRNA sequencing and spatial reconstruction, a global signature of zonation in mouse liver has been established^5^. Immunohistochemical analyses have confirmed the zonal distribution of metabolic enzymes, such as cytochrome P450^13,14^, alcohol dehydrogenase^15^, fatty acid-binding protein^16^, glucose-6-phosphatase and glutamine synthetase^17^ in human liver. Relevant questions, such as the link of metabolic zonation to underlying morphogens, a global assessment of zonation and the underlying regulatory mechanisms and not least also the relation of rodent findings to human liver^18^ are as yet not fully addressed^19^.

In an effort to contribute to a better understanding of these problems, we generated a spatially resolved transcript and DNA methylation map of human hepatocytes, demonstrate an epigenetic zonation in human liver and provide pathway and transcription factor signatures along the porto-central axis that help to understand the underlying programs of liver metabolism, regeneration and tissue structure.

## Results

### Laser microdissection of hepatic zones from human biopsies

**Fig. 1:**
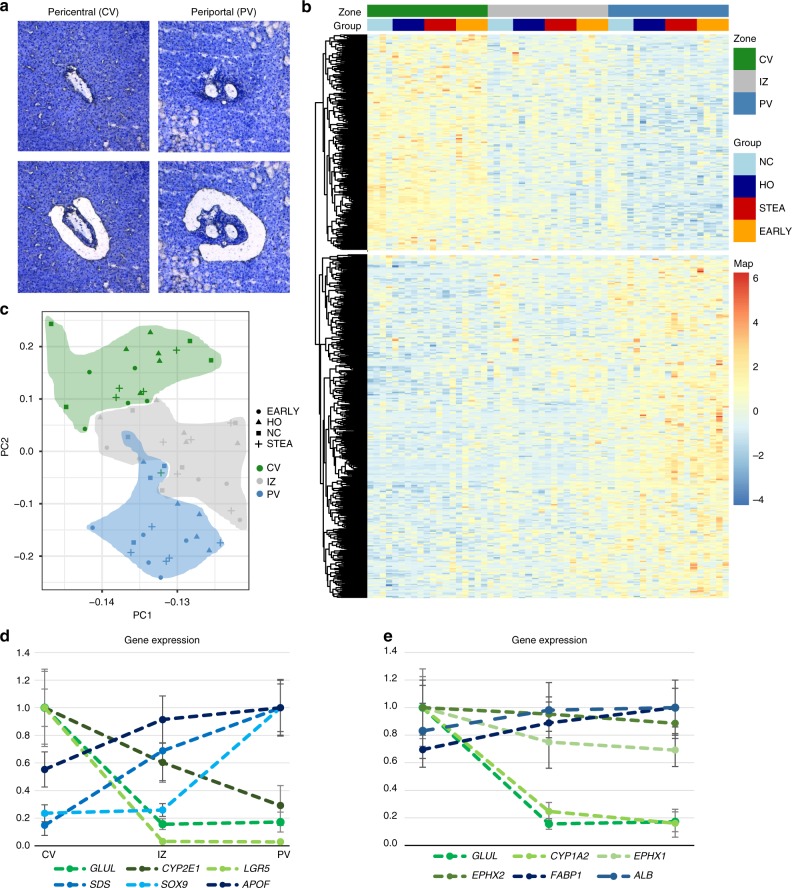
Transcriptional zonation along the porto-central axis. **a** Representative image of laser microdissection of pericentral (CV) and periportal (PV) hepatocytes before LCM (upper images) and after LCM (lower images). **b** Principal component analysis of the 1000 most variable genes. Pericentral (PC) is coloured in green, the intermediate zone (IZ) in red and periportal (PV) in blue. Symbols correspond to phenotypic groups. **c** Expression *z*-scores of zonated genes at pericentral, intermediate and periportal zones (*N* = 19). Zonated genes (805) were determined using edgeR with |log2FC| > 1 and FDR < 0.01 between pericentral and periportal samples and clustered by correlation. The annotation legend displays controls (NC) in light blue, healthy obese (HO) in dark blue, steatosis (STEA) in red and early NASH (EARLY) in orange. **d** Normalized expression (including standard deviation) of genes with previously described zonation in mouse liver in human pericentral, intermediate and periportal zones. Dashed lines are for visualization only. **e** Normalized expression (including standard deviation) of genes previously described as zonated in human liver in the CV, IZ and PV zones, based on immunohistochemistry and in situ hybridization for *GLUL*, *ALB*^17^, *EPHX*, *CYP1A2*^22^, *CYP1A2*^14^ and *FABP*^14^. Dashed lines are for visualization only

Hepatocytes were isolated by LCM from surgical liver samples from 19 patients. Control liver samples (NC, *N* = 4) were obtained from patients undergoing liver resection for metastasis surgery or surgical liver biopsy for the exclusion of malignancy. *N* = 15 liver samples were obtained from patients undergoing bariatric surgery and were categorized based on standardized liver histology employing the NAS score^20^ into phenotypic groups of healthy obese (HO, *N* = 5), bland steatosis (STEA, *N* = 5) and early non-alcoholic fatty liver disease (EARLY, *N* = 5). An overview of the phenotypic characteristics of the patients is provided in Table 1. Liver tissue was shock frozen directly in the operating room ensuring ex vivo times of less than 40 s in all cases. Hepatocytes were isolated by LCM from the pericentral, intermediate and periportal areas of each patient from cryosections stained by cresyl violet (Fig. 1a, Supplementary Figure 1). RNA quality and quantity was assessed with a Bioanalyzer (Agilent, Santa Clara, CA) yielding a mean RIN of 8.09 and a mean yield of 5.43 ng (Supplementary Figure 2A). Samples were subjected to a modified SMART-Seq RNA sequencing protocol^7,21^. Comparisons of the expression of indicator transcripts from cell types, such as immune cells, endothelial cells and fibroblasts indicated an insignificant contamination by non-parenchymal cells (Supplementary Figure 2B, C). Adjacent cryosections were used to generate matching reduced representation bisulfite sequencing (RRBS) data using the CpG methylation insensitive restriction enzymes AluI and HaeIII to cover ~14 million CpGs per sample^8^.

**Table 1:** Summary of analyzed samples

	Control (NC)	Healthy obese (HO)	Steatosis (STEA)	Early NASH (EARLY)
*N*	4	5	5	5
Male %	25	20	40	40
BMI	26 (22–27)	41 (32–47)	46 (32–62)	51 (40–60)
Age, years	57 (54–62)	36 (29–68)	46 (34–67)	44 (41–58)
Fat content, %	0	0 (0–5)	70 (35–90)	40 (40–50)
Inflammation	0	0 (0–1)	0	1 (1–1)
Fibrosis	0	0	0 (0–1)	0 (0–1)
Ballooning	0	0	0	0 (0–1)
NAS	0	0	3 (1–3)	3 (2–4)

The number of patients in each phenotypic category is provided together with demographic and histologic characteristics. All numeric traits are shown as the median with the range provided in parenthesis. BMI, body mass index; NAS, NAFLD activity score^20^

### Signatures of transcriptional and epigenetic zonation

Principal component analysis of the 1000 most variable transcripts (Fig. 1b) revealed highly significant correlations of PC1 and PC2 with hepatic zones (PC1: *r* = 0.47, *p* = 2.0 × 10^−4^; PC2: *r* = 0.86, *p* < 2.2 × 10^−16^, Supplementary Figure 3) indicating zonation as a major driver of variation between samples. Phenotypic group, sex, age, BMI and diabetes state were used as covariates for paired differential analysis using edgeR (FDR < 0.01, |log2FC| > 1). Using the full dataset, we identified 317 genes predominantly expressed in the pericentral zone and 488 genes with predominant periportal expression (Supplementary Data 1). We did not observe genes with highest expression in the intermediate zone, but rather transcriptional gradients from pericentral to intermediate to periportal zone or vice versa (Fig. 1c). Upon analysis of differential expression of steatotic samples (STEA, EARLY) versus samples with normal liver histology (NC, HO), a significant deregulation of 467 genes was observed (Supplementary Figure 4A, Supplementary Data 2), however largely irrespective of hepatic zones (Supplementary Figures 4B, 5). If zonation analysis was restricted to patients with normal liver histology (NC, HO), reduced power led to the detection of only 470 zonated differentially expressed genes (DEGs, Supplementary Data 3). Comparison of pairwise effect sizes between the analyses revealed highly similar log_2_ fold changes (all *p* < 2.2 × 10^−16^) with pairwise correlations (*r*^2^) ranging from 0.80 to 0.98 (Supplementary Figure 6) indicating that the transcriptional zonation signature is persisting during early phases of NAFLD (Fig. 1b).

The transcriptional zonation of the mouse liver lobule has recently been systematically studied from single cell transcriptomic data defining 9 zones^5^. For an interspecies orthologue comparison of human and mouse data, we merged zones 1–2 of the mouse for a comparison to our human pericentral data, the mouse zones 4–5 to the intermediate zone and the mouse zones 8–9 to the periportal zone. We obtained a global transcript correlation of *r*^2^ = 0.77 (*p* < 2.2 × 10^−16^) in periportal and pericentral zones and *r*^2^ = 0.78 (*p* < 2.2 × 10^−16^) in the intermediate zone (Supplementary Figure 7A-C) indicating an overall comparability of the datasets between species. Our zonation comparison was restricted on 313 genes zonated in humans that featured one to one matching orthologues with high confidence expression values in the mouse dataset. We detected 111 genes with conserved zonation signatures between mouse and human (Supplementary Data 4) including highly expressed zonation landmark genes, such as *GLUL*, *CYP2E1*, *SDS* and *APOF* and morphogens like *LGR5* and *SOX9* with low transcript levels (Fig. 1d). However, 202 genes zonated in humans (e.g. Wnt pathway members such as *RSPO2*) were not detected as zonated in the mouse. Most non-corresponding genes in the mouse showed a rather low expression level as compared to our deeply sequenced human data (Supplementary Figure 7D). Comparison of available immunohistochemistry (*CYP1A2*, *EPHX1*, *EPHX2* and *FABP*) and in situ hybridization (*ALB*, *CYP1A2*) data from strongly expressed human genes revealed a similar zonation profile^14,16,17,22^ (Fig. 1e).

**Fig. 2:**
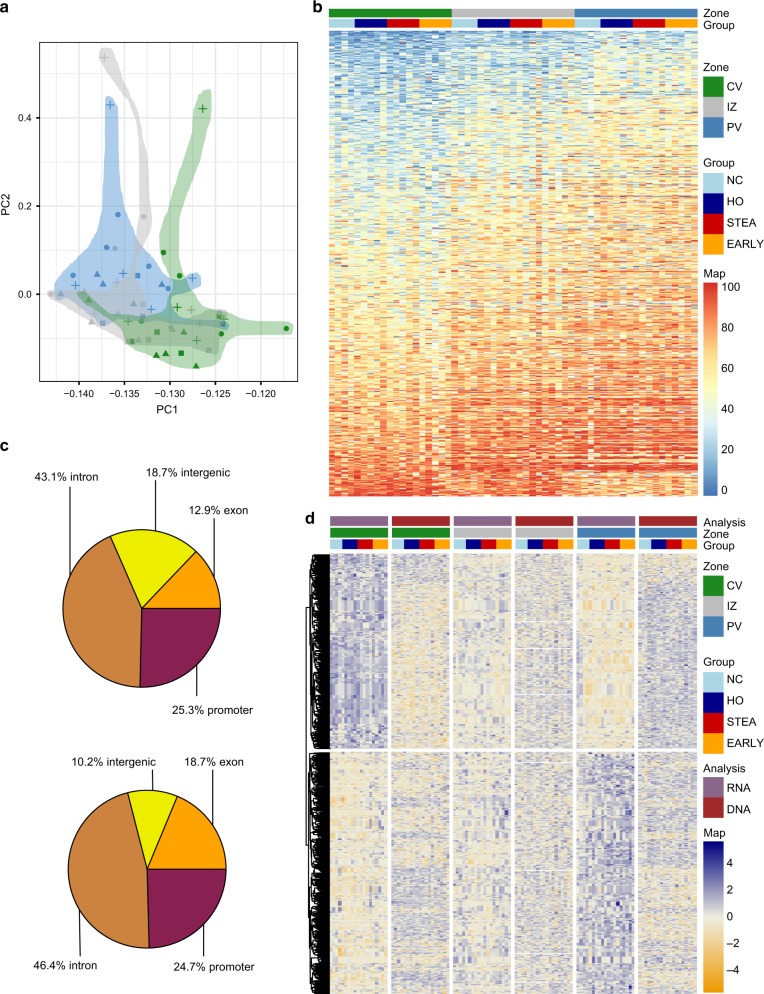
Epigenetic zonation along the porto-central axis. **a** Principal component analysis of the 5000 most variable CpGs (coverage >5 in all samples). Pericentral samples (CV) are coloured in green, the intermediate zone (IZ) in red and the periportal are (PV) in blue. Symbols refer to phenotypic group. **b** Heatmap of methylation values of the top 1000 DMRs (CpG coverage >10, 500 bp tiles, at least 3 CpGs, FDR < 0.01) between pericentral and periportal that were also covered in the intermediate zone (*n* = 19). Average CpG methylation differences between CV and PP range between 12.4% and 49.2%. Blue corresponds to low and red to high average methylation values. The annotation legend displays lean controls (NC) in light blue, healthy obese (HO) in dark blue, steatosis (STEA) in red and early NASH (EARLY) in orange. **c** Genomic annotation of all DMRs (upper pie plot) and of DEG-associated DMRs (lower pie plot). **d** Visualization of gene expression (purple) and DNA methylation (dark red) *z*-scores of DEG-associated DMRs and corresponding genes at pericentral (green), intermediate (red) and periportal zone (blue). In the Heatmap yellow corresponds to low *z*-scores (reduced expression or DNA methylation) and navy to high *z*-scores (increased expression or DNA methylation)

On the epigenetic level, a principal component analysis (Fig. 2a) of the 5000 most variable CpGs (coverage >5 in all samples) showed a separation by hepatic zones with highly significant correlation of PC1 (*p* = 6.7 × 10^−4^, *r* = 0.44, Supplementary Figure 4) and zonation. Following covariate adjustment, a differential methylation analysis (see method for details) between pericentral and periportal samples (MethylKit, CpG coverage >10, 500 bp tiles, at least 3 CpGs, FDR adjusted *p*-value <0.01) yielded 17,862 differentially methylated regions (DMRs) with at least 5% methylation difference (Supplementary Data 5). In contrast we identified only 23 significant DMRs exhibiting the highest methylation difference between PV or CV and IZ. The vast majority of DMRs featured methylation gradients from pericentral to intermediate to periportal zone (Fig. 2b). The majority of DMRs was detected in gene context and only 18.7% of DMRs were located in intergenic regions (Fig. 2c).

To integrate DNA methylation with expression changes, DMRs were annotated to the closest gene transcriptional start site. 44.3% (357) of zone-specific differentially expressed genes were associated with zonal DMRs (1094). A total of 205 DEGs were related with multiple DMRs. DMRs associated to differentially expressed transcripts were predominantly located in introns (46.4%), exons (18.7%) and promoter regions (24.7%, Fig. 2c). DNA methylation differences of significant DEG-associated DMRs and corresponding expression changes (Fig. 2d) were largely anti-correlated (*p* < 2.2 × 10^−16^, *r*^2^ = −0.37). Ninety nine DEGs associated with multiple DMRs displayed a combination of hypo- and hypermethylated patterns of DMRs. We identified 132 upregulated genes associated with 430 hypomethylated DMRs in the pericentral zone and 139 upregulated genes with 291 hypomethylated DMRs in the periportal zone. Based on this clear anti-correlation between DNA methylation and expression gradients, we define these 271 transcripts as epigenetically marked driver genes of zonation (Supplementary Data 6). An example of such potential epigenetically marked driver genes is the Wnt signalling pathway member Palmitoleoyl-Protein Carboxylesterase (*NOTUM*) containing a cluster of six hypomethylated promoter DMRs in the CV zone where the gene is exclusively expressed. Publicly available DNaseI-seq and ChIP-seq data from the hepatocyte-like cell line HepG2 (ENCODE) identifies potential regulatory activity for all seven DMRs (Supplementary Figure 8).

### DNA methylation control at transcription factor binding sites

**Fig. 3:**
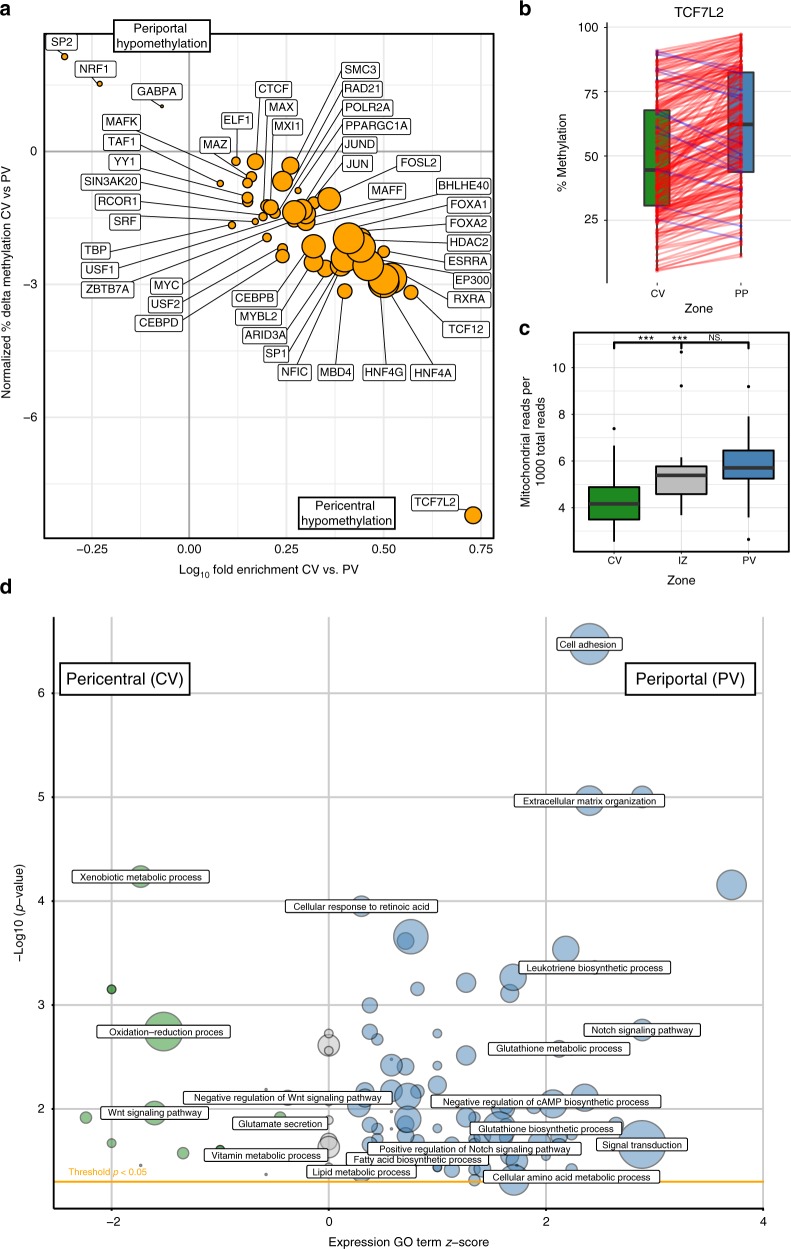
Transcription factor, mitochondrial and metabolic zonation along the porto-central axis. **a** Analysis of methylation of transcription factor binding sites across zones. The *y*-axis depicts the normalized average methylation difference at binding sites overlapping with DMRs for the respective transcription factor. The *x*-axis depicts the log_10_-fold enrichment of DMRs among binding sites. A detailed list and the analysis method are provided in Supplementary Data 7. **b** Methylation of DMRs containing a transcription factor binding site for TCF7L2: Raw methylation data for all sites are shown for the CV and PV regions. **c** Boxplot of the ratio of mitochondrial reads in reduced representation bisulfite sequencing data (*n* = 19) in pericentral (green), intermediate (red) and periportal (blue) zone. Significant differences (*p* < 0.001) are marked by asterisks. **d** Visualization of significantly enriched GO terms (*p* < 0.05) of zonally expressed genes. A negative GO term *z*-score corresponds to GO enrichment in the pericentral zone (green), a positive *z*-score to periportal enrichment (blue). Circle size reflects the number of zonated genes relating to the term. Exemplary GO terms are labelled, while the full list of significant GO terms can be found in Supplementary Data 5

Tissue structure and zonation may be maintained not only by external morphogen gradients, but also by differential signalling efficacy of transcription factors, that may even be uniformly present along the porto-central axis: For instance, the hepatic nuclear factor 4a (HNF4A) is a highly expressed transcription factor (logCPM = 6.12 in our dataset) binding to promoters of ~12% of liver expressed genes^23^. How HNF4A exhibits is differential activity on zonated genes, such as cytochrome P450 and aldehyde dehydrogenase genes^23^ remains unclear. Our RNAseq data—in line with previous reports^24^—show that *HNF4A* expression shows only a marginal trend towards zonation (FDR_PV_CV_ = 0.02, Log_2_FC_PV_CV _= –0.19). Still *Hnf4a* deletion in mice leads to a clear zonal disruption of key metabolic enzymes^25^. Our genome-wide DNA methylation data combined with the ENCODE maps of transcription factor binding sites in HepG2 cells^26^, allowed us to assess a potential epigenetic layer of transcription factor binding control: Indeed 46 of the 59 available transcription factors showed an enrichment of differential methylation at their mapped binding sites in the CV or PV zone (Fig. 3a, *p* < 2.8 × 10^−4^, filtering is described in Methods and TF’s are listed in Supplementary Data 7). Because transcription factors have different preferences for methylated and unmethylated DNA^27^, Supplementary Data 8 summarizes the current literature on binding properties of the analysed TFs combined with DNA methylation in DEEP WGBS HepG2 data to aid interpretation of the zonated TF binding site data. Most of the analyzed and discussed factors show a binding preference to unmethylated DNA. HNF4A (nominal *p* = 4.7 × 10^−300^) binding sites showed the most significant methylation gradient along the porto-central axis. This indicates that epigenetic control may elicit the zonated activity of the otherwise uniformly present transcription factor. The strongest effect size and a pericentral hypomethylation (Δ_methylation_ = 7%, *p* = 1.2 × 10^−80^) was observed for TCF7L2, the key downstream transcription factor of the Wnt signalling pathway^28^. Preferred binding of TCF7L2 at hypomethylated sites indicates higher pericentral signalling efficiency (Supplementary Data 8). An additional analysis using previously identified TCF7L2 binding sites^29^ further supported our zonation results (Supplementary Figure 9). TCF7L2 interacts with HNF4α in multiple hepatocyte specific functions^30^. Like *HNF4α*, overall *TCF7L2* (FDR_PV_CV_ = 0.008, Log_2_FC_PV_CV_ = 0.3) shows only a weak trend towards zonation. Because multiple TCF7L2 protein products with differential activity have been reported^31^, we analyzed alternative splicing of TCF7L2 using the SALMON parallel inference algorithm^32^. No zonation dependent alternative splicing was detected (Supplementary Figure 10), thus indicating that differential binding efficiency rather than transcript abundance underlies the zonation of this factor. In order to provide a broader assessment of zonated TF binding site methylation, we performed an enrichment analysis using predicted TF sites (Supplementary Data 9), which for instance also revealed a pericentral hypomethylation of HNF4A and TCF7L2 sites.

### Zonated features of metabolic networks

Gene ontology overrepresentation analysis both on the DEG and DMR datasets (Supplementary Data 10, 11) recapitulated the proposed distribution of functional activities along the porto-central axis^10^. We find a clear enrichment of GO terms associated with metabolism of steroids, cytochrome P450, xenobiotics or retinoic acid in the pericentral area, glutathione and amino acid metabolism in the periportal area (Fig. 3d). Metabolic zonation is also seen in the GO analysis on the epigenetic level (Supplementary Data 4). A consistent pattern of hypomethylation and overexpression for key zonated metabolic enzymes (Supplementary Data 6) suggests that they are epigenetically marked driver genes controlling zonated regulatory pathways. In the pericentral area we find a strong expression and hypomethylation of *CYP1A2* (logFC = −4.0, 12 DMRs: Δ_meth_ 5.4–33.8%) and *ALDH3A1* (logFC = −5.4, two DMRs: Δ_meth_ 6.3–19.3%). The location of these enzymes corresponds to the pericentral location of liver damage in alcoholic liver disease and Paracetamol toxicity. Periportal candidate epigenetically marked driver genes include glutaminase 2 (*GLS2*, logFC = 3.0, 5 DMRs: Δ_meth_ −15.1, −26.0%) and histidine ammonia-lyase (*HAL*, logFC = 5.1, 3 DMRs: Δ_meth_ −17.2, −33.2%), which are relevant in the glutamate and amino acid metabolism.

When assessing the underlying layer of transcriptional regulation, strong zonation of DNA methylation of binding sites of transcription factors of metabolic relevance (e.g. HNF4A and HNF4G) was evident (Fig. 3a, Supplementary Data 7). Notably, methylation of binding sites for Retinoid X receptor alpha (RXRA), the heterodimeric partner for nuclear signalling of key metabolic transcription factors PPARA, LXRA and FXR^33^, is strongly zonated (*p* = 6.4 × 10^−298^). Similar to *HNF4A*, *RXRA* itself is highly expressed (Log_2_CPM = 6.22) and not zonated (FDR_PV_vs_CV _= 0.97). The ensuing zonation of signalling efficacy of these transcription factors along the porto-central axis has implications for ligands such as free fatty acids which play a key role in the pathogenesis of non-alcoholic fatty liver disease (NAFLD). Interestingly, FXR agonists compounds and drugs such as signalling biliary acids (e.g. obeticholc acid^34^) might therefore elicit a zonal therapeutic response, which may allow targeting of the pharmacodynamic effect to the pericentral disease location in NAFLD.

We also observed a significant gradient of mitochondrial DNA copies deduced from the ratio of mitochondrial reads in the genomic coverage of RRBS data. There is significantly less mitochondrial DNA in the pericentral zone than in the intermediate and periportal zone (*p* = 4.1 × 10^−6^, Fig. 3c). This is in correspondence with a metabolic porto-central oxygen gradient^35^.

### Morphogen gradients and putative stem cell niche

**Fig. 4:**
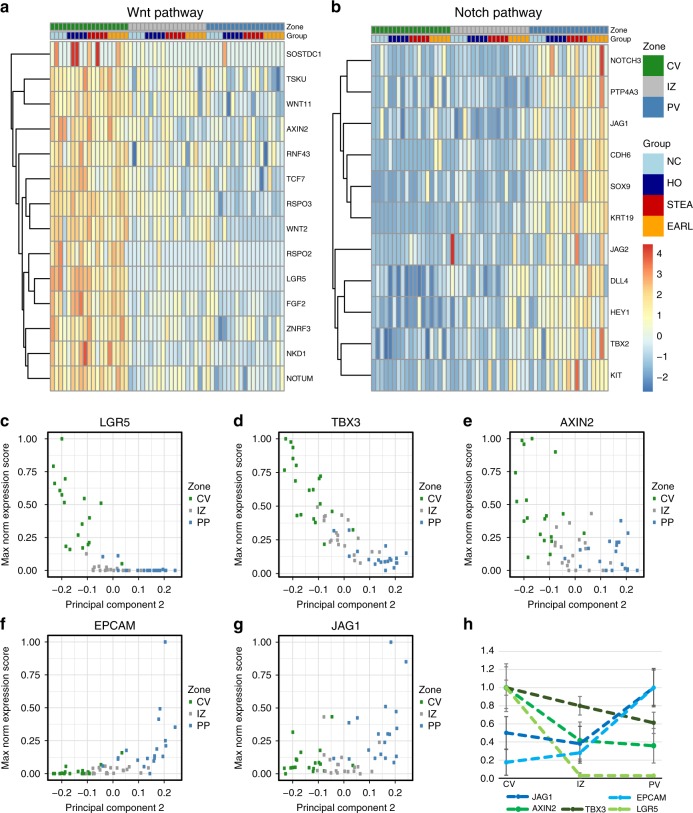
Morphogen gradients and regulatory networks along the porto-central axis. **a** Expression *z*-scores of Wnt signalling pathway genes (GO:0016055, GO:0090263, GO:0030178) show a gene-specific zonation signature. **b** Expression *z*-scores of Notch signalling pathway genes GO:0007219. **c**–**g** Zonation profile of transcripts for morphogens *LGR5*, *AXIN2*, *TBX3*, *EPCAM* and *JAG1*. The *x*-axis is the principal component 2, as a measure for zonation (Note, for visualization reason the PCA2 axis was inverted). **h** Direct normalized gene expression (including standard deviation) of the morphogens depicted in **c**–**g** dissected from the pericentral, intermediate and periportal zones. Dashed lines are for visualization only

The transcriptional and epigenetic zonation includes a series of known morphogen factors (Fig. 3d, Supplementary Data 9, 10). In the periportal zone we find an enrichment for Notch signaling pathway related genes, while there was an enrichment of Wnt signalling among the pericentrally upregulated genes (e.g. GO:0016055, *p* = 5.7 × 10^−4^). A key role of Wnt signaling for the maintenance of liver structure and regeneration is well established from murine liver^12,27–30^. A recent analysis indicated that Wnt pathways are also involved in human liver zonation^19^. Our deep integrated datasets allowed a deeper look into the putative role of pericentral Wnt signalling networks in human liver homoeostasis: On both, the epigenetic and transcriptional level, we identify a composed network of regulatory changes affecting selected members of the pathway but not a global upregulation or activation (Fig. 4a). Prominent examples are the epigenetically marked driver loci *AXIN2* (Δ_methCV_PV_ = 5.0–29.3%), *NOTUM* (Δ_methCV_PV_ = 10.7–36.5%), and *LGR5* (Δ_methCV_PV_ = 9.2%). In contrast, classical Wnt agonists such as *WNT3A*, *WNT3* and genes of the export machinery (e.g. porcupine, *PORCN1* and Wntless, *WLS*) are not zonally expressed, suggesting that the pericentral WNT gradient in hepatocytes might not primarily be regulated by them. Negative regulators of Wnt signaling such as *NOTUM*, *NKD1*, *RNF43* and *ZNRF3* are upregulated 1 to 2 log levels pericentrally creating a tightly controlled signalling environment around hepatocytes of this zone. The Wnt signalling enhancers *RSPO2*, *RSPO3* and *LGR5* are expressed 2–7 log levels higher pericentrally than periportally suggesting sensitization of pericentral hepatocytes for canonical Wnt signals^29^. Interestingly, *LGR5* (logFC = 7.15, FDR = 4.88 × 10^−26^) shows the highest fold change of all significantly DEGs. Finally, binding sites of the main Wnt-driven transcription factor (T-cell factor 7, *TCF7L2*, *p* = 1.22 × 10^−80^) in the liver^28^ are >5 fold pericentrally enriched among the pericentral DMRs (Fig. 3b, c). We conclude that morphogen gradients are controlled by multi-layered transcriptional and epigenetic Wnt signalling signatures in the zonated parenchyma.

The pericentral overexpression and hypomethylation of *LGR5* (Fig. 4c) and *AXIN2* (logFC = 2.24, FDR = 6.17 × 10^−5^, Fig. 4e) is strongly correlated to the expression of the early liver progenitor gene *TBX3* (pairwise expression correlation to *AXIN2*: *r*^2^ = 0.51, *p* = 5.36 × 10^−5^ and *LGR5*: *r*^2^ = 0.86, *p* < 2.2 × 10^−16^). *TBX3* is the transcript with the strongest zonation (logFC_PV_CV_ = 2.26, FDR = 4.1 × 10^−59^) and highest expression in the pericentral area (Fig. 4d). *TBX3* is embedded in a landscape of 11 pericentrally hypomethylated DMRs (ΔmethCV_PV = 6.9–43.6%). Together all our findings are consistent with the hypothesis that pericentral *AXIN2* and *LGR5* positive cells^36,37^ are also in humans a source of regeneration under steady-state conditions as reported from murine lineage tracing experiments^38^.

Also on the periportal side, we identify epigenetically marked driver genes such as the NOTCH ligand jagged 1 (*JAG1*, Fig. 4g) (log_2_FC = 2.03, FDR = 1.64 × 10^−5^, ΔmethCV_PV = −5.3 to 10.0%)^39^ and find a clear enrichment for a Notch GO term (GO:0007219) associated with periportal DEGs (*p* = 1.68 × 10^−4^, Fig. 4b). The liver stem cell marker *EPCAM*^40–42^ showed a particularly strong periportal expression (log_2_FC = 5.42, FDR = 1.5 × 10^−9^, Fig. 4f) potentially marking the periportal hepatic progenitor cell (HPC) niche^39,43^. Interestingly, pathways of other liver-related morphogens such as FGF, TGF beta, HGF and BMP are neither epigenetically nor transcriptionally zonated at an FDR < 0.01 level, underlining the importance of the Wnt and Notch pathways (Fig. 4h) for the maintenance of liver structure under steady-state conditions.

## Discussion

The systematic generation of reference epigenomes and transcriptomes through the ENCODE and IHEC projects allows deep insights into the functional state of isolated cells or tissues. The integration of cell-specific epigenomic and transcriptomic profiles with positional information appears as an essential next step to better understand the organisation of organs and tissues and to dissect regional program alterations in disease. Here, we present the first spatially resolved map of human hepatocyte genomic architecture by integrating methylome and transcriptome data. Previous human data is heterogenous and patchy and has used either measurements of enzyme activities^15,44,45^ or immunohistochemistry and in situ hybridization assays targeting whole-gene families. We excluded the activity assays from our confirmatory comparisons in Fig. 1e, because enzyme activities reflect often multiple transcripts, cofactor concentrations, phosphorylation state and other confounders. While confirming previous immunohistochemical and gene expression studies^14,16,17,19,22^, we identify hundreds of additionally zonated transcripts and observe intriguing correlations between transcription and DNA-methylation.

We optimized a pipeline starting from fast sampling in the operating room, followed by a documented LCM on sectional stains, which were directly used for the generation of genome wide transcriptome and RRBS data^8^ from 100 to 200 captured cells per section. This approach is complementary to the alternative single cell and FACS sorting approaches as it provides greater sequencing depth and uses the pathological classification and direct zonal localisation within the tissue. Single cell approaches on the other hand while providing a cleaner view on cellular heterogeneity they have to rely on computational modelling of their spatial position using independent molecular signatures as anchor points. This highly informative single cell modelling usually comes at the expense of sequencing depth and gene coverage. We believe that our approach is an ideal complementation to single cell approaches as it provides strong sequencing depth, positional information and allows to comprehensively cover the epigenetic dimension.

Such positional datasets can be queried in multiple ways offering a new and integrated approach of epigenetic and transcriptional mechanisms controlling zonation, liver function and morphogen control: First, we demonstrate for example, that epigenetic zonation underlies the transcriptional porto-central gradient and that anti-correlated DNA methylation and transcription may define a set of potentially epigenetically marked driver genes, that form a deeper layer underlying the zonation gradients. Second, we propose that strongly zonated DNA-methylation at transcription factor binding sites may form an additional layer of epigenetic zonation control. Differential methylation of binding sites may indicate a spatial activity of otherwise uniformly expressed transcription factors, such as HNF4A and RXR. Drugs targeting RXR (e.g. obeticolic acid) or its interaction partner PPARα (i.e. fibrates) are likely to preferentially affect target genes in pericentral hepatocytes, which corresponds to the disease location of metabolic liver disease as a zone 3 disorder^20^. Third, the integrated genomic dataset also allows to assess the existing concepts on liver regeneration onto the human steady-state situation: We demonstrate a strong transcriptional and epigenetic gradient of Wnt signalling and modulating factors. The pericentral expression of *LGR5* and *AXIN2* and the corresponding gradient of the liver progenitor marker *TBX3*^46,47^ indicate a pericentral source of hepatocyte regeneration^38^ under steady-state conditions in humans. Conversely, in the portal area, we observed a *NOTCH* and *EPCAM* signature. This may correspond to hepatic progenitor cells at the border to the biliary tree, which may form a relevant source of regeneration under injury conditions^36,39,48^. Expansion of this compartment clearly needs to be confirmed in further studies using cirrhotic human liver. In our dataset, we see that zonation in both—the transcriptomic and epigenetic- dimensions is the by far dominant signature and remains largely preserved in steatosis and early NASH. This finding is compatible with the complete regeneration and remodelling capacity of human liver in early NAFLD.

In summary our study opens a new path for a functional dissection of parenchymal differences in the liver lobule connecting positional information to known aspects of liver biology. Many of the observations described will be helpful for a deeper understanding of liver organ function in relation to disease.

## Methods

### Human liver samples

Liver samples were obtained intraoperatively in patients in whom an intraoperative liver biopsy was indicated on clinical grounds such as during scheduled liver resection, exclusion of liver malignancy during major oncologic surgery, or assessment of liver histology during bariatric surgery. Standardized histology by a single pathologist (C.R.) employed the NAFLD activity score (NAS)^20^. Samples were frozen immediately in liquid nitrogen ensuring an ex vivo time of less than 40 s in all cases. Patients with evidence of viral hepatitis, hemochromatosis or alcohol consumption >20 g per day for women and 30 g per day for men were excluded. All patients provided written informed consent. The study protocol was approved by the institutional review board (Ethikkommission der Medizinischen Fakultät der Universität Kiel, D425/07, A111/99), before study commencement.

### Laser capture microdissection

The frozen tissue was cut in 20 µm slices and mounted on precooled glass-slides. Immediately after mounting, the tissue was fixed by incubation in 70% EtOH for 2 min and stained by incubation in a cresyl violet 50% EtOH solution for 30 s with two short subsequent wash steps in 70% EtOH, followed by a 2 min air-drying step. All steps were performed on ice. In sum, an area of 500,000 µm^2^, from of 5 to 10 periportal fields and intermediate zones or hepatozytes surrounding 15–20 central veins per sample, was micro-dissected using a ZEISS PALM MicroBeam LCM in Auto-LPC mode. Tissue pieces for RRBS were frozen and maintained at −80 °C. Samples for RNA-Seq were immediately processed.

### RNA seq

After microdissection, RNA was isolated using the RNeasy Micro Kit (Qiagen, Hilden, Germany). Concentration and RNA Quality were obtained by using an Agilent BioAnalyzer (Agilent, Santa Clara, CA). The typical yield was between 4.5 ng and a RIN between 8 and 9. The RNA was reverse transcribed and amplified with the SMARTer Universal Low Input RNA Kit for Sequencing (Takara, Clontech) and 1 ng of RNA. Libraries for next generation sequencing were constructed with the Nextera DNA Library Prep Kit (Illumina, San Diego, Ca, USA) and sequenced on a HiSeq2500 Sequencer.

### RRBS

Reduced representation bisulfite sequencing (RRBS) of micro-dissected cells was performed as previously described^8^ with modifications. Briefly, cell lysis of micro-dissected material was performed directly in the caps of the capturing tubes by treatment with 8 µl lysis buffer (10 mM Tris-HCl, 5 mM EDTA) and 2 µl proteinase K (1 mg per ml, NEB) at 55 °C for 3 h. For proteinase inactivation, 0.5 µl Pefabloc (21 mM, Sigma) was added for 1 h at room temperature. Restriction was performed using the CpG methylation insensitive enzymes HaeIII (25 U, NEB) and AluI (5 U, NEB) supplemented with 1.5 µl CutSmart buffer (10X, NEB), 1 µl MgAce (5 mM) and 1 µl yeast tRNA (100 ng) at 37 °C for 18 h. Enzymes were inactivated at 80 °C for 20 min. A-Tailing was achieved with 1 µl Klenow Fragment (3′ → 5′exo−, 5 U per µl, NEB) and 1 µl dATP (10 mM, NEB) at 37 °C for 30 min followed by enzyme inactivation at 75 °C for 20 min. Methylated Illumina TruSeq sequencing adapters (adapter 1:ACACTCTTTCCCTACACGACGCTCTTCCGATCT, adapter 2:GATCGGAAGAGCAC ACGTCTGAACTCCAGTCAC) were ligated using 1 µl pre-annealed adapters (100 µM), 0.5 µl T4 Ligase (2,000,000 U per µl, NEB) and 2 µl ATP (10 mM, NEB) at 16 °C for 22 h. Subsequent bisulfite conversion was performed using the EZ DNA Methylation Gold Kit (Zymo). Adapter ligated fragments were amplified by PCR using 0.5 µl each of indexed TruSeq primers (10 µM, Primer I5: AATGATACGGCGACCACCGAGATCTACAC[i5]ACACTCTTTC CCTACACGACGCTCTTCCGAT, Primer I7:GATCGGAAGAGCACACGTCTGAACTC CAGTCAC[i7]ATCTCGTATGCCGTCTTCTGCTTG), 3 µl PCR Buffer (10X, Qiagen), 1.2 µl MgCl_2_ (25 mM), 2.4 µl dNTPs (10 mM) and 0.5 µl Hot Start Taq (5 U per µl, Qiagen) in a 30 µl reaction with 95 °C for 15 min, 22 cycles of 95 °C for 30 s, 60 °C for 30 s, 72 °C for 1 min and final elongation at 72 °C for 7 min. After purification with 0.8 X Ampure XP Beads (Beckman Coulter) libraries were sequenced on a HiSeq2500 (Illumina) using TruSeq SBS Kit v3 – HS chemistry in a single read run with 90 bp read length.

### Data processing

Reads were trimmed with Trim Galore! (v0.4.2) http://www.bioinformatics.babraham.ac.uk/projects/trim_galore/) for adapter contamination and 3′-ends with base quality below 20. RRBS reads were trimmed in RRBS mode. The reads were then aligned to the 1000 genomes version of the human GRCh37 reference and further processed as described below. RRBS reads were aligned with the BWA (v0.6.2)^49^ wrapper methylCtools (v0.9.2)^50^. Samtools (v1.3)^51^ and Picard tools (v1.115) (http://broadinstitute.github.io/picard/) was utilized for converting, merging and indexing of alignment files. Bis-SNP was used for SNP (dbSNP, v138, http://www.ncbi.nlm.nih.gov/SNP) aware realignment, quality recalibration and methylation calls. Differentially methylated sites were detected using MethylKit (v1.3.1)^52^ with adjustment for the potential confounders phenotype, sex, age, BMI, donor and the prevalence of diabetes (Supplementary Fig. 3). The tool was modified to treat missing sites as zero-covered sites in the aggregation step of the tiled analysis. After merging both strands the calls were filtered to sites with at least 10X coverage. We searched for differentially methylated 500 bp tiles (step-size: 500 bp) with at least 3 CpGs, a maximum *q*-value of 0.01 and minimal methylation difference of 5%. Mitochondria, X and Y chromosomes were excluded. The resulting DMRs were annotated to closest genes and genomic features (promoter, exon, intron or intergenic) using Gencode gene models (v19)^53^ with the help of bedtools (v2.20.1)^54^.

RNA-seq reads were aligned with the STAR (v2.5.2a)^55^ and the per sample 2-pass mapping strategy processing all reads in both passes as described in the documentation. Read counts were summarized to Gencode gene models (v19)^53^ with featureCounts (v1.5.0-p3)^56^ counting primary alignments only. EdgeR (v3.16.5) was used to detect differentially expressed genes with maximal *q*-value of 0.01 and minimal absolute logFC of 1. We discarded genes for which fewer than five samples had a counts per million value above 0.5, calculated normalization factors and robustly estimated the dispersion. In our default setting we used a model adjusting for phenotype, sex, age, BMI, donor and the prevalence of diabetes (Supplementary Figure 3).

### Integrative analysis and visualization

For interspecies comparisons, reconstructed mouse zones 1 and 2 were considered as pericentral, zones 4 and 5 as intermediate and zones 8 and 9 as periportal. Spearman rank statistics of sequencing depth normalized mRNA expression levels (log(CPM + 1)) were calculated for mouse single cell data^5^ and human LCM-RNAseq data with one-to-one matching orthologs. The zonation comparison was restricted to genes with high confidence expression values corresponding to a CPM > 0 in the mouse dataset after background subtraction as previously described^5^. For visualization of exemplary genes across the three zones, expression data were normalized to the zone with the highest expression for each individual gene. Potential epigenetically controlled driver genes were defined as upregulated genes that also featured at least one hypomethylated DMR annotated to the gene. Genomic tracks of expression and methylation data (bigwig) were generated using bedtools^54^ and UCSC tools^57^ and visualized in the IGV browser^58^. GO term enrichment analysis of DEGs and DMR associated genes was performed with DAVID^59^ and significant DEG GO terms (*p* < 0.05) were visualized using GOplot^60^.

For analysis of methylation of transcription factor binding sites with available information of the hepatocyte cell line HepG2 in ENCODE (V3), a 2 × 2 table was constructed for each transcription factor as follows: The 196,621 methylation regions (500 bp intervals), were categorized in regions with and without binding sites for the respective transcription factor and by differential methylation between the CV and PV zones as analysed in the main DMR analysis (Supplementary Data 6) yielding four categories (noDMR.noTFBS, noDMR.TFBS, DMR.noTFBS and DMR.TFBS). Deviation from the null hypothesis (i.e. equal distribution of binding sites between DMR and regions without differential methylation) was tested by Fisher's exact test. The methylation gradient between CV and PV for intervals without and with binding sites for the respective transcription factors yields the zonated methylation difference Delta.TXF. Because all DMRs show a mild porto-central demethylation gradient and this gradient is reflected in DMRs with transcription factor binding sites, the zonated differential methylation is normalized to the mean of Delta.TXF (−1.27%) for the final normalized Delta.TXF.Norm.

## Electronic supplementary material

Supplementary Information

Supplementary Data 1

Supplementary Data 2

Supplementary Data 3

Supplementary Data 4

Description of Additional Supplementary Files

Supplementary Data 5

Supplementary Data 6

Supplementary Data 7

Supplementary Data 8

Supplementary Data 9

Supplementary Data 10

Supplementary Data 11

## Data Availability

The data generated in this study is available at the NCBI Gene Expression Omnibus (GEO) (http://www.ncbi.nlm.nih.gov/geo) under identifier GSE105127.
