# Supplementary material for: Epigenomic map of human liver reveals principles of zonated morphogenic and metabolic control
Source: Nat Commun. 2018 Oct 8;9:4150. doi: 10.1038/s41467-018-06611-5 (PMC6175862; doi:10.1038/s41467-018-06611-5)
Supplement: Supplementary file 1 — Supplementary Information [file 41467_2018_6611_MOESM1_ESM.docx]

Epigenomic map of human liver reveals principles of zonated morphogenic and metabolic control

Brosch, Kattler et al.

**Supplementary Information**

**Supplementary Figures**


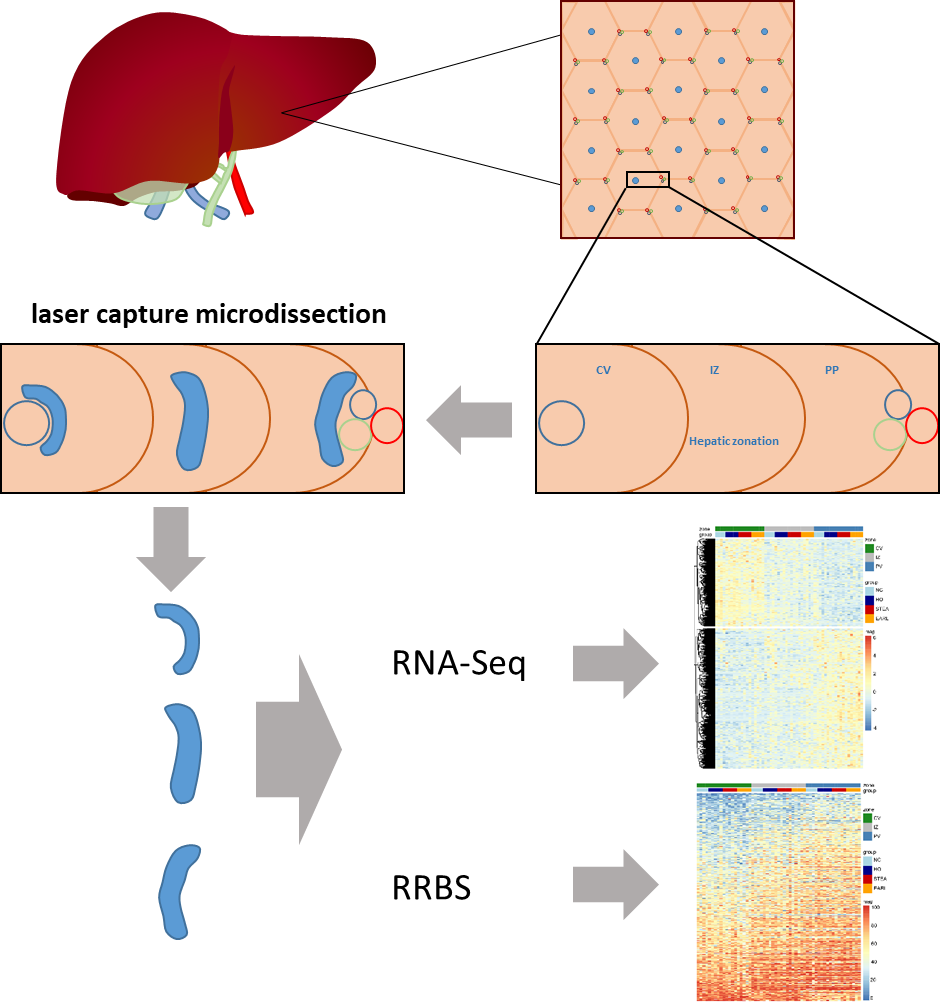


***Supplementary Figure 1: Schematic representation of the workflow used in this study.*** Liver samples were obtained intraoperatively in patients in whom an intraoperative liver biopsy was indicated Laser capture microdissection for the periportal, intermodal and pericentral with subsequent RRBA and RNA-Seq was performed as described in Material and Methods.


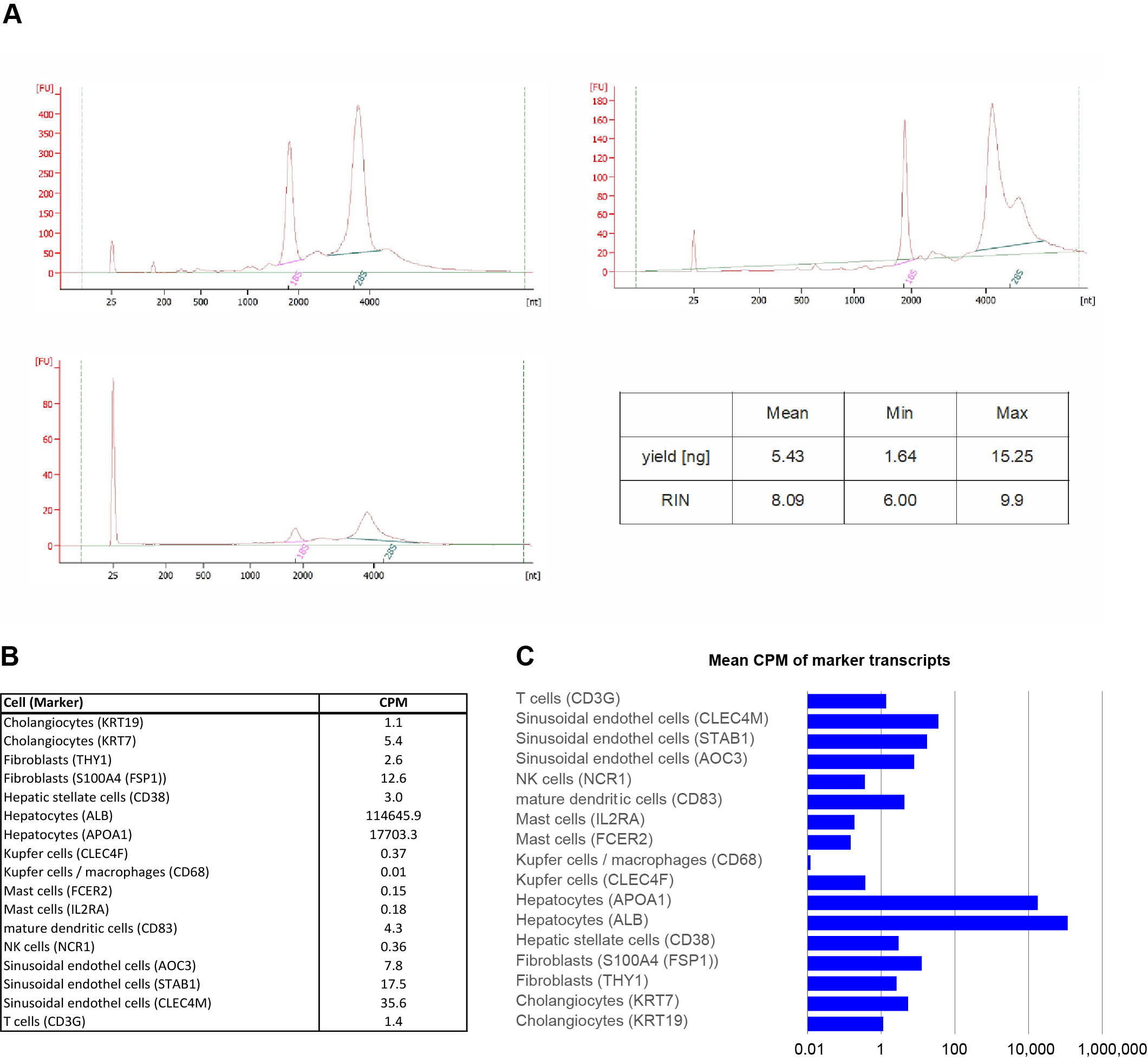


***Supplementary Figure 2: RNA quality of LCM tissue and contamination with non-hepatocyte RNA. A:*** Agilent Bioanalyzer electropherograms of RNAs obtained from LCM microdissected tissue from a representative sample from the pericentral, intermediate and periportal areas. ***B and C:*** Tabular (B) and graphical (C) comparison of the RNA abundance of indicator transcripts of non-parenchymal cells (NPCs). The data indicate a non-significant contamination by NPCs as the respective NPC indicator transcripts are at least two orders of magnitude less abundant than the hepatocyte indicator transcripts of albumin and APOA1.

**A**

| **all expressed genes** | | **Variance [%]** | **Zone** | **Phenotype** | **Gender** | **Age** | **BMI** | **Diabetes** |
| --- | --- | --- | --- | --- | --- | --- | --- | --- |
| PC1 | correlation | 96.43 | 0.259442 | 0.0293952 | 0.090155 | 0.138816 | 0.075797 | 0.080151 |
|  | p value |  | 0.05131 | 0.8282 | 0.5048 | 0.3031 | 0.5752 | 0.5534 |
| PC2 | correlation | 1.71 | 0.01977 | 0.2873835 | 0.058432 | 0.044387 | 0.195218 | 0.27646 |
|  | p value |  | 0.8839 | 0.03019 | 0.6659 | 0.743 | 0.1456 | 0.03737 |
| PC3 | correlation | 0.53 | 0.493319 | 0.02491101 | 0.104782 | 0.117822 | 0.066651 | 0.172074 |
|  | p value |  | 9.66E-05 | 0.8541 | 0.4379 | 0.3827 | 0.6223 | 0.2006 |
|  |  |  |  |  |  |  |  |  |
| **1000 most variable genes** | | **Variance [%]** | **Zone** | **Phenotype** | **Gender** | **Age** | **BMI** | **Diabetes** |
| PC1 | correlation | 90.27 | 0.472986 | 0.7059 | 0.178412 | 0.096588 | 0.081507 | 0.004124 |
|  | p value |  | 2.03E-04 | 0.05108663 | 0.1842 | 0.4748 | 0.5467 | 0.9757 |
| PC2 | correlation | 1.51 | 0.861013 | 0.2716978 | 0.2792 | 0.113683 | 0.144704 | 0.27653 |
|  | p value |  | 2.20E-16 | 0.04091 | 0.03544 | 0.3998 | 0.2828 | 0.03732 |
| PC3 | correlation | 0.94 | 0.016878 | 0.2131011 | 0.024856 | 0.034053 | 0.463568 | 0.226954 |
|  | p value |  | 0.9008 | 0.1115 | 0.8544 | 0.8014 | 0.000282 | 0.08956 |

**B**

| **all CpGs** | | **Variance [%]** | **Zone** | **Phenotype** | **Gender** | **Age** | **BMI** | **Diabetes** |
| --- | --- | --- | --- | --- | --- | --- | --- | --- |
| PC1 | correlation | 98.46 | 0.155953 | 0.07216958 | 0.083147 | 0.371824 | 0.097793 | 0.201514 |
|  | p value |  | 0.2467 | 0.5937 | 0.5386 | 0.004401 | 0.4693 | 0.1328 |
| PC2 | correlation | 0.06 | 0.237859 | 0.3308178 | 0.590151 | 0.239649 | 0.013707 | 0.477551 |
|  | p value |  | 0.0748 | 0.01195 | 1.36E-06 | 0.07258 | 0.9194 | 0.000172 |
| PC3 | correlation | 0.05 | 0.256088 | 0.1284953 | 0.172686 | 0.41087 | 0.218177 | 0.107495 |
|  | p value |  | 0.05451 | 0.3408 | 0.199 | 0.0015 | 0.103 | 0.4261 |
|  |  |  |  |  |  |  |  |  |
| **5000 most variable CpGs** | | **Variance [%]** | **Zone** | **Phenotype** | **Gender** | **Age** | **BMI** | **Diabetes** |
| PC1 | correlation | 88.23 | 0.437238 | 0.0253262 | 0.287092 | 0.080826 | 0.120156 | 0.070141 |
|  | p value |  | 0.000672 | 0.8517 | 0.03037 | 0.5501 | 0.3733 | 0.6041 |
| PC2 | correlation | 0.88 | 0.270612 | 0.3596026 | 0.579609 | 0.332019 | 0.011268 | 0.559276 |
|  | p value |  | 0.04175 | 0.006008 | 2.31E-06 | 0.01163 | 0.9337 | 6.12E-06 |
| PC3 | correlation | 0.50 | 0.262819 | 0.2036444 | 0.384315 | 0.111199 | 0.271693 | 0.44539 |
|  | p value |  | 0.04825 | 0.1287 | 0.003162 | 0.4102 | 0.04091 | 0.000517 |

***Supplementary Figure 3: Correlation of zonation and phenotype to the epigenomic signature. A:*** Correlation of zonation and phenotypic parameters in the expression data: The top 1000 most variable genes (median log(CPM+1) = 1.75) and all expressed genes (median log(CPM+1) = 1.60) showed comparable expression levels. ***B:*** Correlation of zonation and phenotypic parameters in the methylation data:


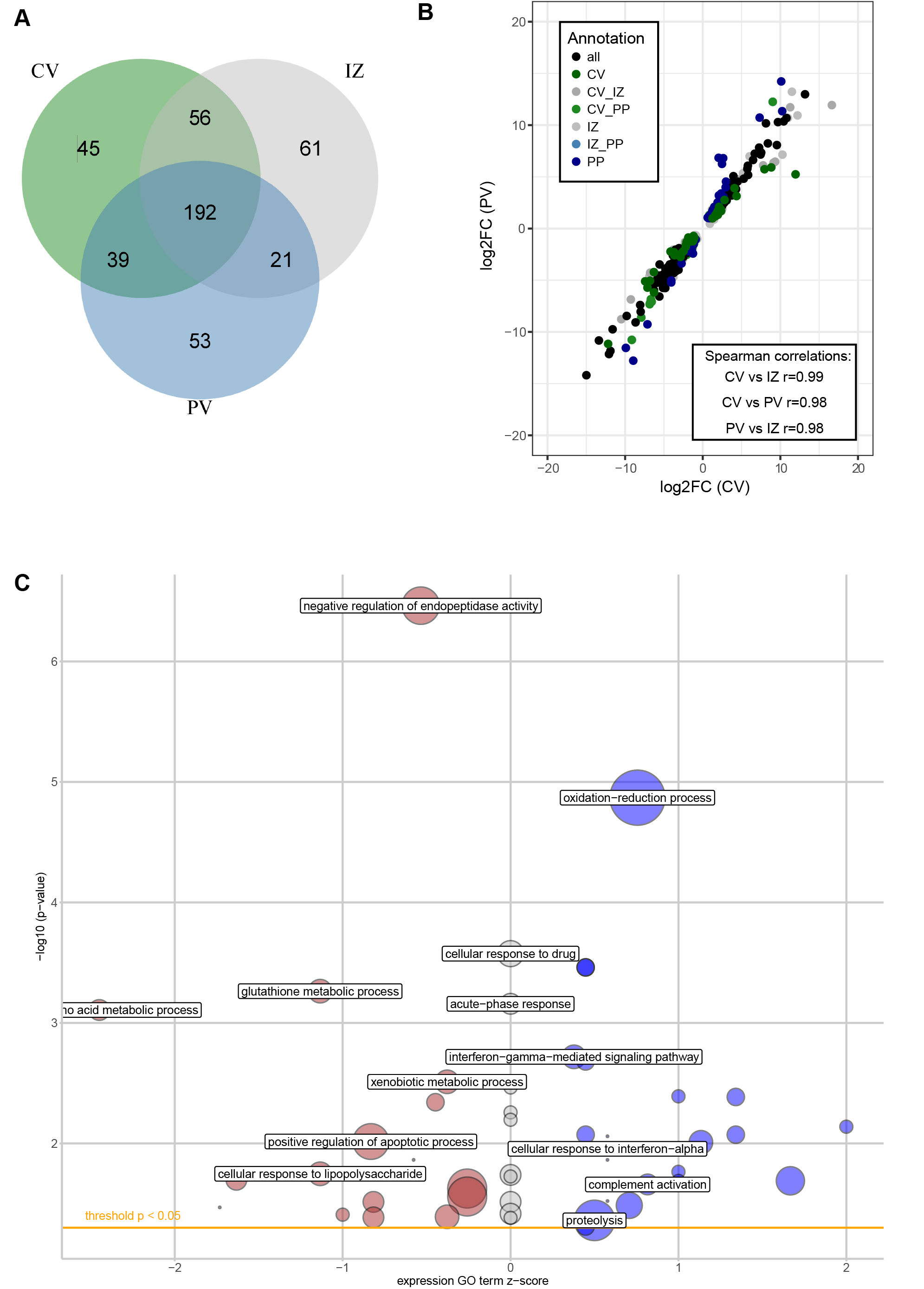


***Supplementary Figure 4: Differential gene expression in steatosis. A:*** Differentially expressed genes (DEGs) between steatotic samples (bland steatosis and early NASH, n = 10) and samples with normal liver histology (normal lean controls and healthy obese controls, n = 9) were determined using edgeR with |log2FC| > 1 and FDR < 0.01 separately for the pericentral (CV), intermediate (IZ) and periportal (PV) zones. The Venn diagram depicts the overlap of significant DEGs between the three zones. ***B:*** Scatterplot of pericentral versus periportal log2 fold changes of genes significantly deregulated in steatosis in any of the three zones with corresponding Spearman correlations between zones indicate preserved zonation of phenotypically differentially expressed transcripts. ***C:*** Visualization of significantly enriched GO terms (p<0.05) of genes deregulated in steatosis. A negative GO term z-score illustrates downregulation in steatosis (red), a positive z-score corresponds to upregulation (blue). Circle size reflects the number of zonated genes relating to the term and exemplary GO terms are labelled. The onset of NAFLD is may thus be marked by a beginning upregulation of inflammatory signatures accompanied by a downregulation of metabolic functions across all zones.


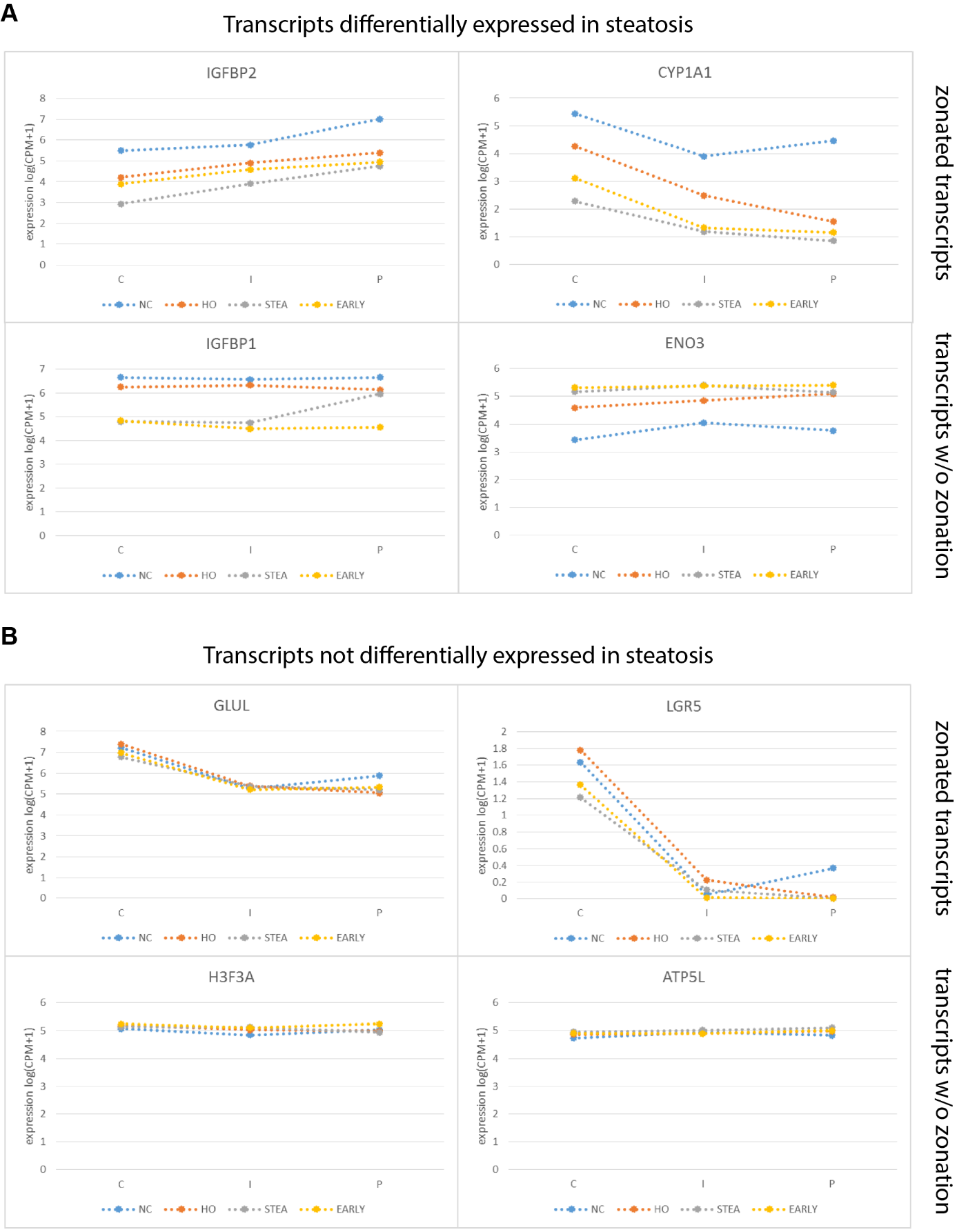


***Supplementary Figure 5: Zonation is not affected by the steatotic phenotype.*** The Figure provides more detailed visualization of the zonal expression of transcripts, that are (***A***) or are not (***B***) differentially expressed in steatosis. It is evident (as in Supplementary Figure 3B), that the differential expression affects all zones and that the zonation gradient is maintained in steatosis.


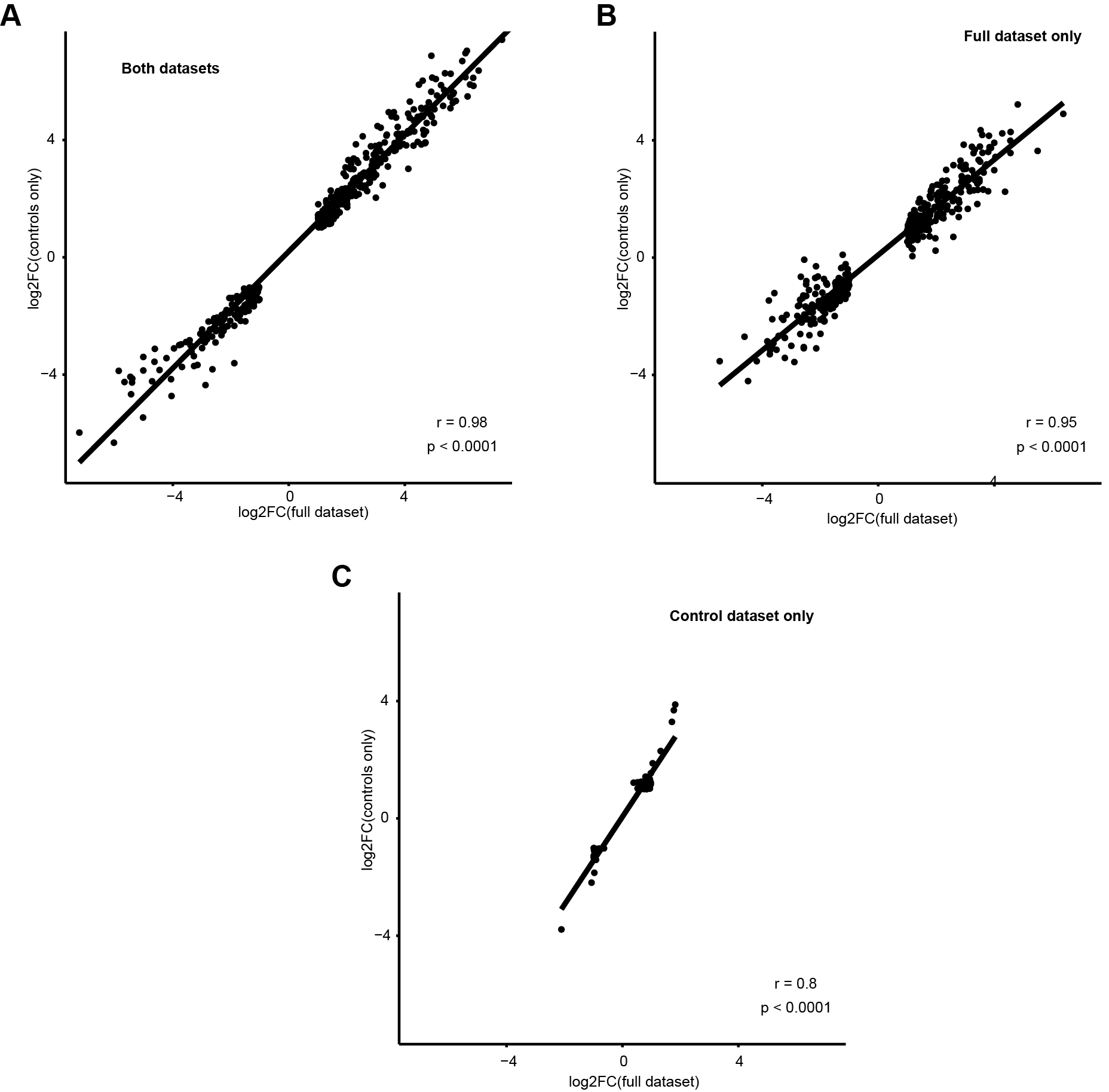


***Supplementary Figure 6: Discovery in full dataset or controls only.*** Comparison of transcriptional zonation in the full dataset (including steatosis and early NASH samples) versus controls only (normal lean controls and healthy obese controls). ***A:*** - Scatterplot of log2 fold changes of zonated genes detected in both comparisons (Spearman correlation between full dataset and controls only: r = 0.98, p<2.2e-16). ***B:*** Scatterplot of log2 fold changes of DEGs uniquely detected in the full dataset (Spearman correlation between full dataset and controls only: r=0.95, p<2.2e-16). ***C:*** Scatterplot of log2 fold changes of DEGs uniquely detected in controls (Spearman correlation between full dataset and controls only: r = 0.80, p<2.2e-16).


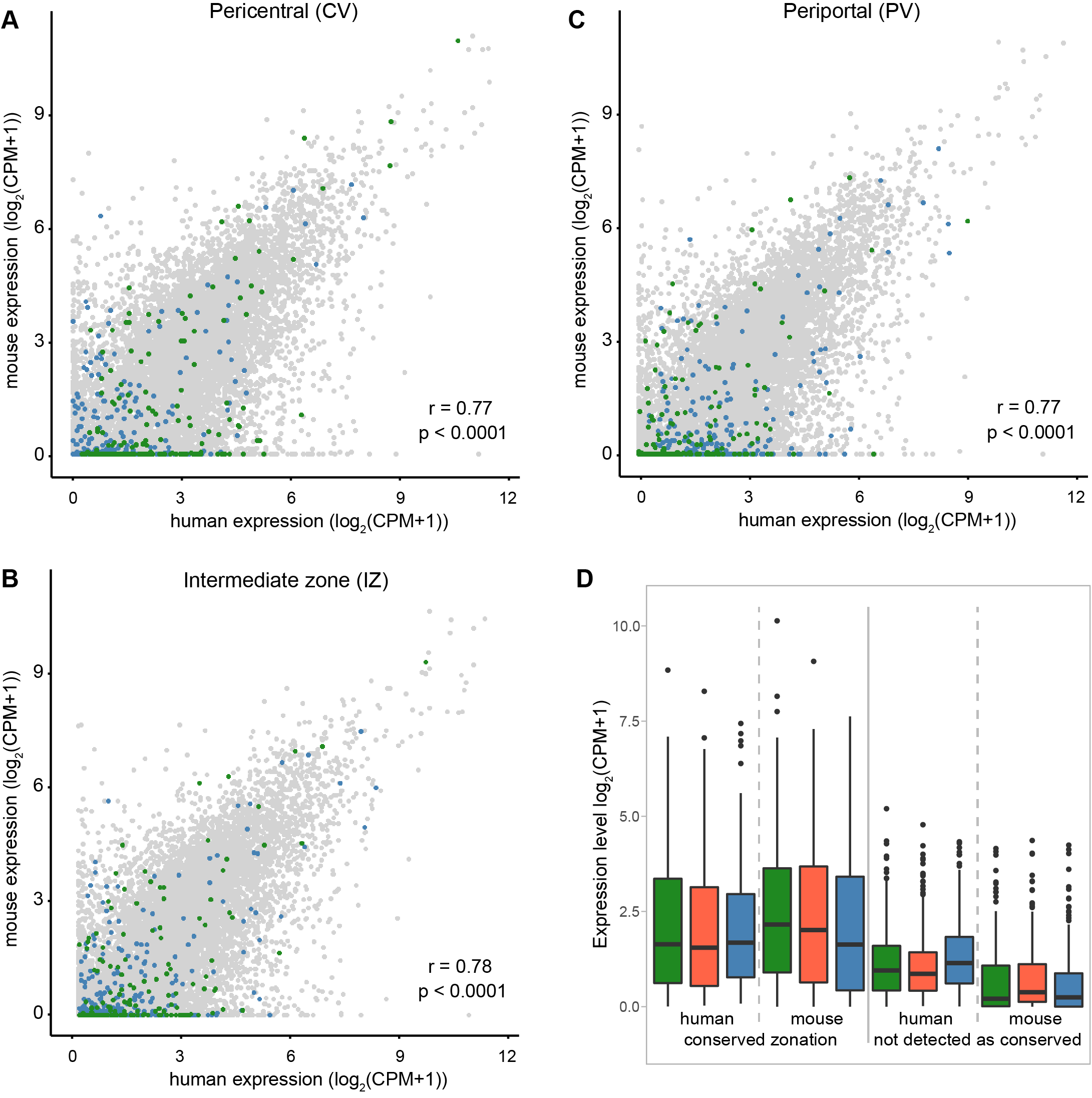


***Supplementary Figure 7: Comparison of mouse and human liver zonation. A-C***: Scatter plots with Spearman rank statistics of mRNA expression levels of mouse single cell data ^5^ and human LCM-RNAseq data with one-to-one orthologs. Reconstructed mouse zones 1 and 2 were considered as pericentral (A), zones 4 and 5 as intermediate (B) and zones 8 and 9 as periportal (C). Genes with significant zonation in human (|logFC|>1, FDR < 0.01) are highlighted in green (pericentral) and blue (periportal). ***D:*** Boxplot of expression levels of genes with conserved zonation pattern (111 genes) and of genes zonated in human, but not detected as zonated in mouse (202 genes) in pericentral (green), intermediate (red) and periportal (blue) zones.


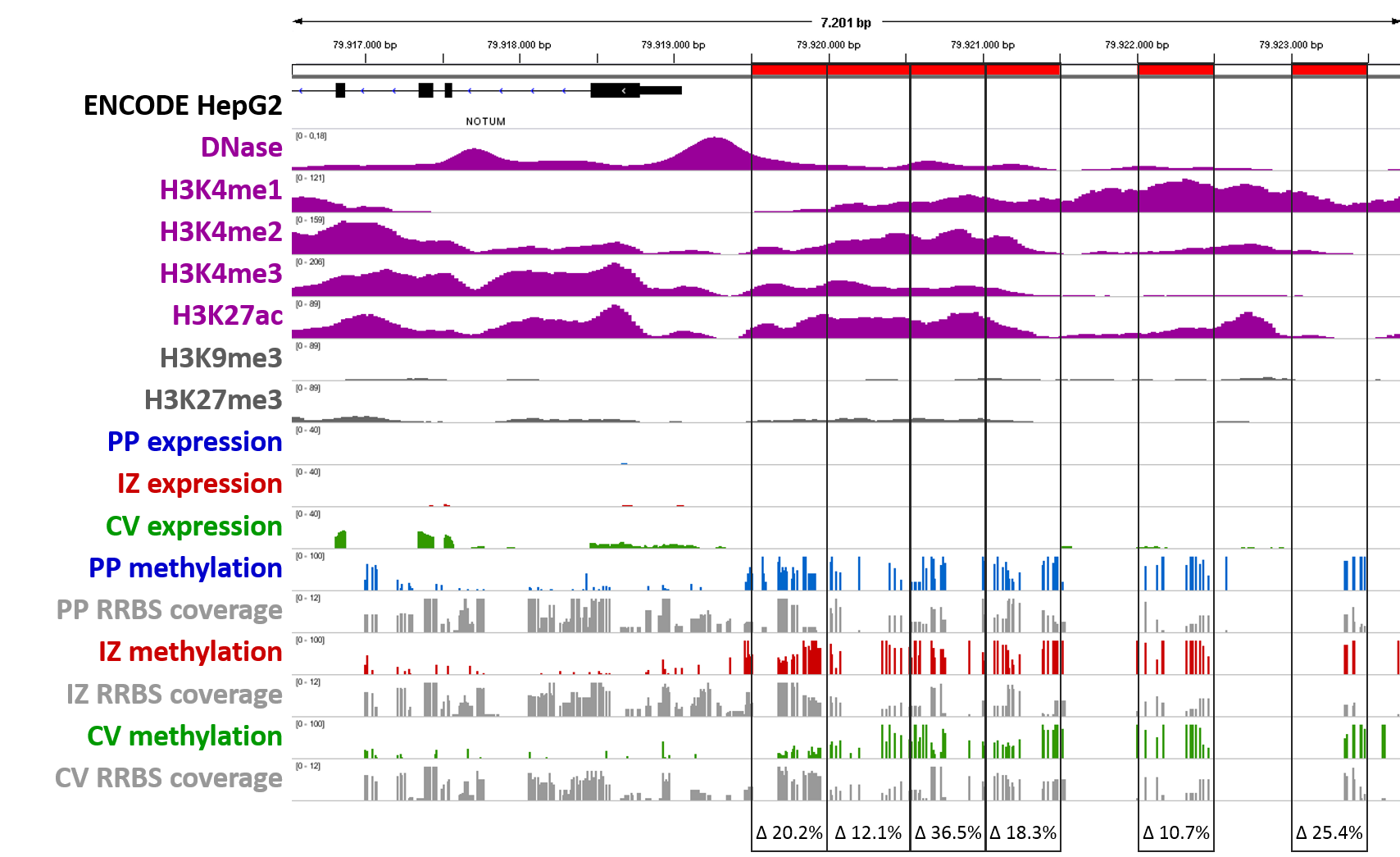


***Supplementary Figure 8: Anti-correlated expression and DNA methylation at an exemplary potential epigenetic driver gene.*** Genome browser view of the pericentrally expressed Wnt signaling pathway member Palmitoleoyl-Protein Carboxylesterase (NOTUM) with 6 DMRs in the 5 kb promoter region. An exemplary sample (HO) was used for visualization of expression and DNA methylation. Pericentral is colored in green, intermediate in red and periportal in blue. RRBS coverage tracks are shown in grey. Encode data of the hepatocyte-like cell line HepG2 (DNase, H3K4me1, H3K4me2, H3K4me3 and H3K27ac displayed in purple, H3K9me3 and H3K27me3 in grey) are also included for visualization of potential regulatory regions. DMRs are framed by black boxes. The displayed percentages are mean methylation difference between pericentral and periportal cells calculated from all samples (n = 19) considering only CpGs with coverage of at least 10.


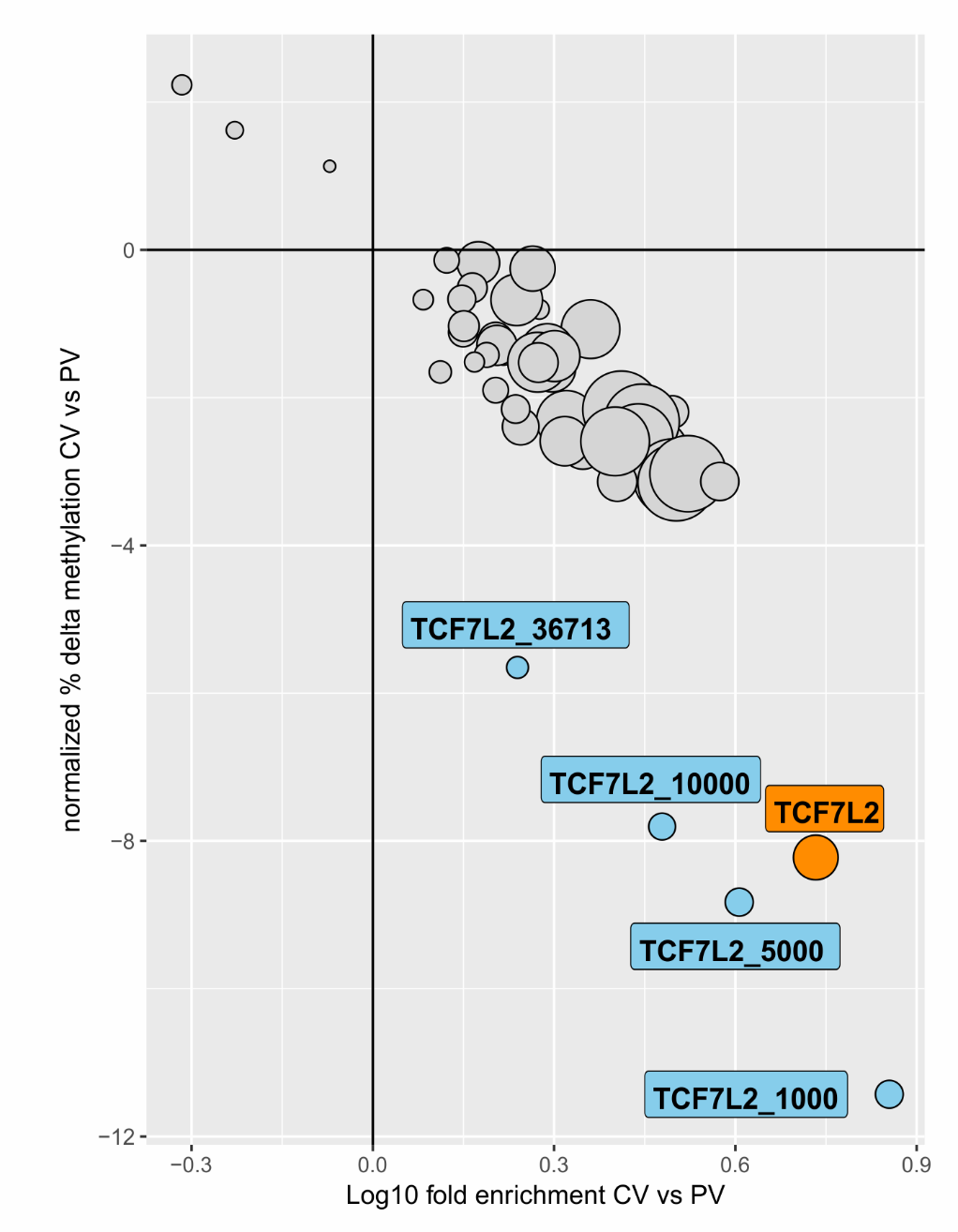


***Supplementary Figure 9: Validation of TCF7L2 enrichment analysis***. Analysis of methylation of transcription factor binding sites across zones as described in Figure 3 of the main manuscript. The y-axis depicts the normalized average methylation difference at binding sites overlapping with DMRs for the respective transcription factor. The x-axis depicts the log10-fold enrichment of DMRs among binding sites. In addition to the data shown in Figure 3, TCF7L2 binding sites identified by Wang et al. (2013, PMID 23769673) were used in the differential methylation analysis of TCF7L2 binding sites (blue) and compared to the analysis in this study (orange). TCF7L2 binding sites were ranked by p-value and all (denoted as TCF7L2_36713), top 10.000 (denoted as TCF7L2_10000), top 5000 (denoted as TCF7L2_5000), top 1000 (denoted as TCF7L2_1000) were plotted in blue. Indeed, as filtering of sites described by Wang et al. is more stringent, stronger zonated enrichment is observed, thus further strengthening this finding.


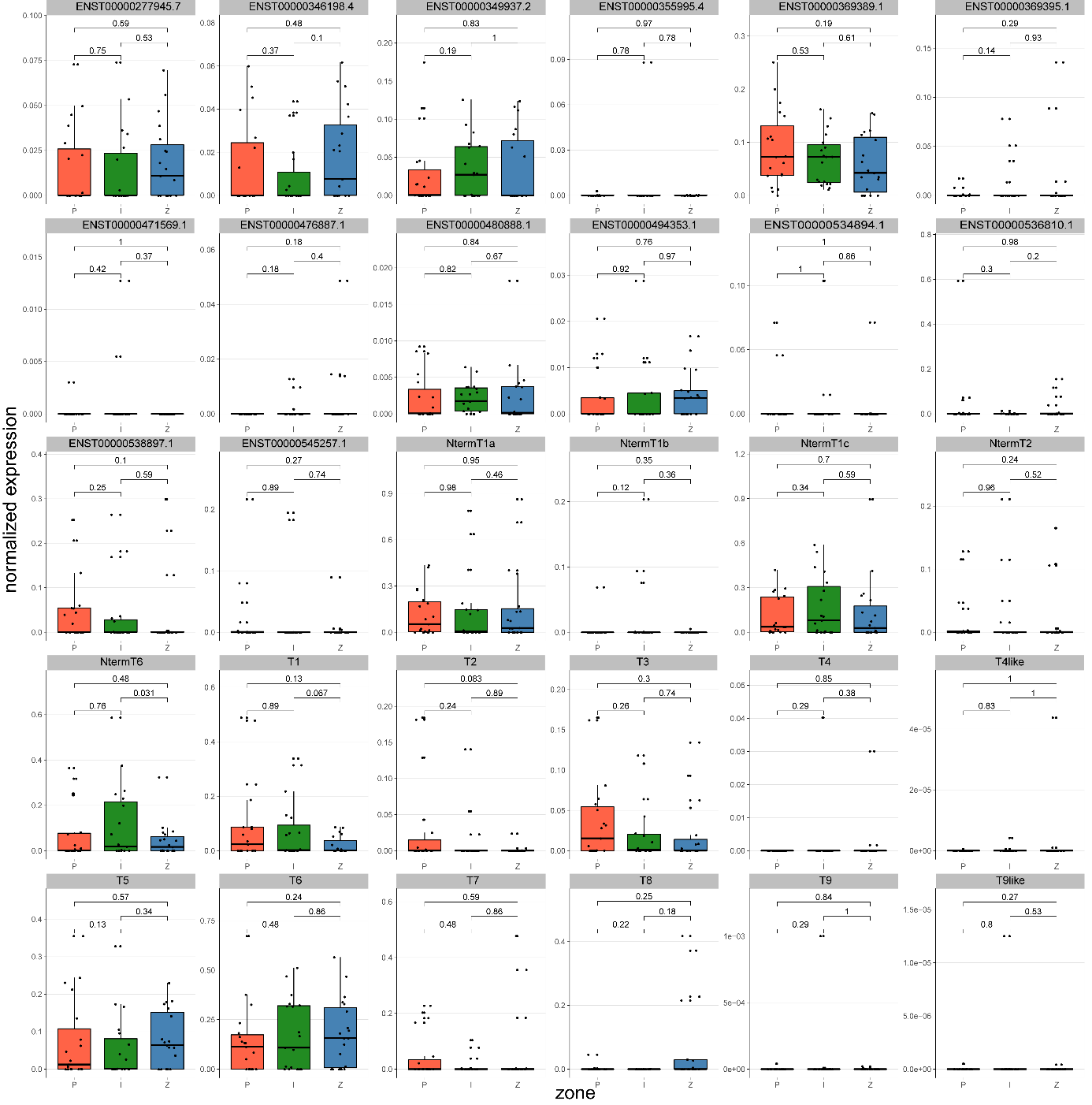


***Supplementary Figure 10: Analysis of alternative splice site usage in TCF7L2.*** TCF7L2 is reported to have a multitude of alternative transcripts (Neve 2017, PMID 24463962). To analyze alternative splicing of the TCF7L2 transcripts in a zonation dependent manner, all annotated ENSEMBL transcripts in addition with the newly identified transcripts in Neve 2017 were considered. Reads matching to the genomic region of TCF7L2 were extracted from the genome wide alignment file and realigned using the Salmon parallel inference algorithm (Patro et al. 2017 PMID 28263959). Transcripts with matching reads in at least 3 samples were further analyzed. None of the analyzed transcripts showed a significantly (Wilcox test) different expression in the three zones (P,I,Z).
