# Supplementary material for: Epigenomic map of human liver reveals principles of zonated morphogenic and metabolic control
Source: Nat Commun. 2018 Oct 8;9:4150. doi: 10.1038/s41467-018-06611-5 (PMC6175862; doi:10.1038/s41467-018-06611-5)
Supplement: Supplementary file 6 — Description of Additional Supplementary Files [file 41467_2018_6611_MOESM6_ESM.pdf]

Description of Additional Supplementary Files

File Name: Supplementary Data 1
Description: Differential methylation in all samples: Differentially methylated regions (DMRs) were analyzed with MethylKit (adjustment for the potential confounders phenotype, gender, age, BMI, donor and the prevalence of diabetes , 500 bp tiles with at least 3 CpGs and coverage ≥ 10, FDR < 0.01 and methylation difference ≥ 5 %) and annotated to closest genes. The table contains DMRs detected between portal area (PV) and central area (CV) supplemented with information about the intermediate zone (IZ). It provides genomic coordinates of DMRs, Ensembl gene identifier (Geneid), gene symbol (Symbol), mean methylation difference (meth.diff) and false discover rates (FDR) of the pairwise comparisons between the three zones. In addition, the mean methylation values (methylation) per zone and an annotation of the zone with significantly reduced methylation (hypomethylated) are given. DMRs with insufficient coverage in the intermediate zone are marked as NA in the IZ columns. The list is ordered by mean methylation difference in descending order.

File Name: Supplementary Data 2
Description: Candidate epigenetic driver genes were defined as differentially expressed genes (|log2FC| > 1, FDR < 0.01) between periportal (PV) and pericentral (CV) zone that were associated with differentially methylated regions (DMRs, methylation difference ≥ 5 %, FDR < 0.01) featuring hypomethylation coinciding with a transcriptional upregulation in the respective zone. The table provides Ensembl gene identifier (Geneid), gene symbol (Symbol), the log2 fold changes (logFC) and false discover rates (FDR) between periportal and pericentral expression, the log CPM values for the assessment of transcription level and an annotation of the zone with predominant expression. In addition, genomic coordinates of DMRs, mean methylation difference (meth.diff) and false discover rates (FDR) between zonal methylation levels, an annotation of DMRs to genomic features and an annotation of the zone with significantly reduced methylation (hypomethylated) are given. Candidate epigenetic driver genes are marked in the respective column. The list is ordered by log2 fold change in ascending order.

File Name: Supplementary Data 3
Description: Gene ontology enrichment (p ≤ 0.05) of differentially expressed genes (|log2FC| > 1, FDR < 0.01) between periportal (PV) and pericentral (CV) zone: The table is annotated by the zone with upregulated expression (upregulated) of genes enriched for the respective GO term. It provides GO Terms, description, gene count, percentage of all upregulated genes in the respective zone (%), enrichment p value, genes as Ensemble IDs and fold enrichment. The list is ordered by zone and enrichment p value.

File Name: Supplementary Data 4
Description: Gene ontology enrichment (p ≤ 0.05) of genes associated with differentially methylated regions (DMRs, methylation difference ≥ 15 %, FDR < 0.01) between periportal (PV) and pericentral (CV) zone: The table is annotated by the zone with hypomethylated genes (hypomethylated) enriched for the respective GO term. It provides GO Terms, description, gene count, percentage of all hypomethylated genes (%), enrichment p value, genes as Ensemble IDs and fold enrichment. The list is ordered by zone and enrichment p value.
